# Golden Tea Program: Examining the Feasibility of a Culturally-based, Mindfulness Intervention for Older Chinese

**DOI:** 10.1093/geroni/igaf122.3947

**Published:** 2025-12-31

**Authors:** Keith Chan, Wenting Zhang, Isabel Ching, Rosemary Li, Soko Setoguchi

**Affiliations:** Hunter College, New York, New York, United States; Tea Arts & Culture, Brooklyn, New York, United States; Hamilton-Madison House, New York, New York, United States; Rutgers University, New Brunswick, New Jersey, United States; Rutgers University, Rutgers University, New Jersey, United States

## Abstract

The Golden Tea Program is a 7-week culturally-based mindfulness intervention that aims to improve overall mental health, reduce loneliness, delay cognitive decline, and strengthen a sense of cultural connectedness, conducted with a community-based sample of 32 Chinese older adults in New York City. The intervention is rooted in the study and appreciation of tea by creating the space and time to slow down with intention and experience the beauty of everyday interactions. Drinking tea is a sensory experience that can be appreciated through a multicultural and multigenerational lens. The overall goal is to: 1) Determine the feasibility of this culturally-based mindfulness intervention rooted in tea culture with Chinese older adults, 2) Test the influence of this program to reduce social isolation and loneliness, increase social connections, strengthen cultural identity, delay cognitive decline and improve overall mental health, and 3) Conduct a careful mixed methods evaluation of the proposed intervention using community-based intervention framework. Preliminary findings indicated acceptability and feasibility of the Golden Tea program, with participants reporting improvements in cognition, social well-being and mental health. This pilot study can serve as a demonstration of a feasible, low-risk and low-cost community-based program for Chinese older adults.

